# Respiratory Virus-Induced PARP1 Alters DC Metabolism and Antiviral Immunity Inducing Pulmonary Immunopathology

**DOI:** 10.3390/v16060910

**Published:** 2024-06-04

**Authors:** Mohamed M. Mire, Srikanth Elesela, Susan Morris, Gabriel Corfas, Andrew Rasky, Nicholas W. Lukacs

**Affiliations:** 1 Department of Pathology, University of Michigan, Ann Arbor, MI 48109, USA; 2 Mary H Weiser Food Allergy Center, University of Michigan, Ann Arbor, MI 48109, USA; 3 Department of Otolaryngology, Kresege Hearing Research Institute, University of Michigan, Ann Arbor, MI 48109, USA; corfas@umich.edu

**Keywords:** dendritic cell, cell metabolism, innate immunity, virus infection

## Abstract

Previous studies from our laboratory and others have established the dendritic cell (DC) as a key target of RSV that drives infection-induced pathology. Analysis of RSV-induced transcriptomic changes in RSV-infected DC revealed metabolic gene signatures suggestive of altered cellular metabolism. Reverse phase protein array (RPPA) data showed significantly increased PARP1 phosphorylation in RSV-infected DC. Real-time cell metabolic analysis demonstrated increased glycolysis in PARP1-/- DC after RSV infection, confirming a role for PARP1 in regulating DC metabolism. Our data show that enzymatic inhibition or genomic ablation of PARP1 resulted in increased ifnb1, il12, and il27 in RSV-infected DC which, together, promote a more appropriate anti-viral environment. PARP1-/- mice and PARP1-inhibitor-treated mice were protected against RSV-induced immunopathology including airway inflammation, Th2 cytokine production, and mucus hypersecretion. However, delayed treatment with PARP1 inhibitor in RSV-infected mice provided only partial protection, suggesting that PARP1 is most important during the earlier innate immune stage of RSV infection.

## 1. Introduction

Respiratory syncytial virus (RSV) infects nearly all infants by the age of two and is the leading cause of bronchiolitis in children worldwide [1]. In 2017 alone, the global healthcare cost of RSV infection in children under five years of age was estimated to be over $5 billion [2]. While RSV is especially detrimental in infants whose airways are small and easily occluded, RSV has also been widely recognized as an important pathogen in transplant recipients, patients with chronic obstructive pulmonary disease (COPD), the elderly, as well as other patients with chronic lung disease, especially asthma [3,4]. Several studies have revealed the prevalence of RSV reinfections, demonstrating the ability of the virus to suppress antiviral immune responses needed to drive long-lasting immunity [5,6,7]. Previous studies from our laboratory have established the myeloid dendritic cell (DC), a significant contributor to host acquired immunity, as a key innate cell during RSV that drives infection-induced pathology [8,9]. A significant body of research points to RSV manipulating the host cell immune responses, especially DC function [10,11,12]. This creates a particularly important paradigm with DC, with evidence from our studies suggesting that the latter play a role in driving changes in cell metabolism that regulates cell immune function through multiple pathways that now include PARP1.

Poly (ADP-ribose) polymerases, also known as PARPs, are a group of closely related enzymes capable of transferring ADP-ribose from NAD+ molecules to cellular targets. PARP-mediated utilization of NAD+ results in a net loss of the pyridine nucleotide moiety which necessitates the resynthesis of NAD+ to replete cellular pools. These enzymes play crucial roles in a multitude of cellular processes including transcription, translation, and, most critically, DNA repair [13,14,15,16,17,18]. Of the 17 members of the PARP family, PARP1, PARP2, and PARP5a/b can deposit poly ADP-ribose units (PARylation) while the rest catalyze mono ADP-ribosylation. PARP1 is the most abundant member of the family and accounts for the vast majority of cellular PARylation [19,20,21]. Overactivation of PARP1 has been shown to result in up to 50–80% reduction in cellular NAD+ levels, suggesting the high potential of PARP1 in regulating the cellular metabolism of immune cells [22,23]. NAD is an important co-factor in a variety of metabolic pathways, particularly glycolysis.

Immunometabolism is an area that has proven to be central to how immune cells regulate their function and impact the direction of antipathogen responses. Innate cells have been shown to increase glycolytic metabolism upon activation [24]. Glycolysis, though less efficient at producing ATP per molecule of glucose, is a quicker process and can provide the rapid bursts of energy immune cells require. Glycolysis also generates metabolic intermediates that can be used for biosynthesis, helping immune cells such as the DC mount an effective response. Data generated in our laboratory have begun to address metabolic changes in DC that are altered by and during RSV infection responses. Most interestingly, analysis of reverse phase protein array (RPPA) data which identifies phosphorylated cytoplasmic proteins has revealed that PARP1 exhibits, in relative terms, the most activation (phosphorylation) in RSV-infected DC.

## 2. Materials and Methods

### 2.1. Mice

Six-to-eight-week-old female C57BL/6J and BALBc mice were purchased from The Jackson Laboratory (Sacramento, CA, USA). Parp1-/- mice (129S background) were gifted by Dr. Gabrial Corfas (University of Michigan). The University of Michigan University Committee on Care and Use of Animals reviewed and approved all animal experiments.

### 2.2. PARP1 Inhibitor

Mice were administered with 0.33 mg/kg of Talazoparib (Selleckchem-BMN 673) via oral gavage. In vitro studies utilizing bone-marrow-derived dendritic cells (BMDC) were given Talazoparib (0.2 µM) for 30 min prior to RSV or mock infection.

### 2.3. RSV Infection

RSV line 19 (Subgroup A) was used for all experiments. Line 19 is a clinical RSV isolate derived from an infected individual at the University of Michigan Children’s Hospital [25,26]. Virus stocks were propagated using Hep-2 cells and viral concentration were determined using plaque assays. Animals were subjected to intratracheal installation of 2.5 × 10^5^ plaque forming units.

For DC transfer experiments, dendritic cells were grown from 8-week-old WT and Parp1-/- mice and cultured for 6 days in the presence of 10 ng/mL of recombinant murine granulocyte macrophage-colony stimulating factor (Gm-CSF; R&D Systems). These non-adherent cells were infected with RSV for 4 h and then washed thoroughly with cold PBS. As previously described [27], BMDC were then intratracheally installed into the lungs of naïve WT mice which were themselves then infected with RSV.

### 2.4. Histology

Mice right lungs were perfused with formaldehyde and then embedded in paraffin. Lung sections were stained with periodic acid-Schiff (PAS) or hematoxylin/eosin (H/E). A Zeiss Axio Imager Z1 with AxioVision 4.8 software (Zeiss, Munich, Germany) collected photomicrographs.

### 2.5. Lymph Node Restimulation

Lymph nodes were enzymatically dispersed with collagenase A 1 mg/mL (Roche, Indianapolis, IN, USA) and 20 U/mL DNase I (Sigma, St. Louis, MO, USA) in RPMI with 10% FCS and further dispersed via an 18 gauge needle (10 mL syringe). RBCs were lysed, samples filtered through 100 micron nylon mesh, and cells were then resuspended in PBS. Cells (5 × 10^5^) were plated in 96-well plates and restimulated with 1.5–3.0 ×  10^5^ pfu of RSV. The supernatant was collected after 48 h to measure cytokine protein levels using a customized Bioplex assay (Bio-Rad Laboratories, Hercules, CA, USA).

### 2.6. Flow Cytometry

Mice left lopes were enzymatically dispersed with collagenase A 1 mg/mL (Roche, Indianapolis, IN, USA) and 20 U/mL DNase I (Sigma, St. Louis, MO, USA) in RPMI with 10% FCS and further dispersed via an 18 gauge needle (10 mL syringe). RBCs were lysed, samples filtered through 100 micron nylon mesh, and cells were then resuspended in PBS. Live cells were labeled using LIVE/DEAD Fixable Yellow Dead Cell Stain kit (Thermo Fisher Scientific, Waltham, MA, USA). Surface markers were identified using Abs (clones) against the following antigens, all from BioLegend: anti-Cd11c (N418), MHC II (M5/114.15.2), Cd11b (M1/70), CD103 (2E7), F4/80 (BM8), and Ly6C (HK1.4). For CD11c+ DC: CD11b+CD11c+MHCII+. For interstitial macrophages: CD11b+CD11c-F4/80+. For Ly6C+ monocytes: CD11b+Ly6C+.

### 2.7. Array-Based Omics Data

BMDC were grown from naïve C57BL/6J female mice as previously described [28,29]. Briefly, bone marrow was harvested from mouse long bones to isolate the hematopoietic progenitor cells. Cells were cultured for 10 days in the presence of 10 ng/mL of recombinant murine granulocyte macrophage-colony stimulating factor (Gm-CSF; R&D Systems). BMDC were infected with RSV or media for 24 h prior to microarray transcriptional profiling based on a Mouse Gene ST 2.1 array that was processed in the University of Michigan DNA Sequencing Core using the Affy Plus kit. Differential expression analysis (≥0.6 |log2FC| and FDR ≤ 0.05) was done using the LIMMA package in R [30]. Pathway analysis (difference of class-RSV vs Ctrl; FDR < 10%) was done using the Gene Set Enrichment Analysis (GSEA) software from the Broad Institute [31]. The database of gene expression profiles for cell lines following pharmacologic or genetic perturbation from the LINCS L1000 project was used to identify PARP1 modulated genes. Gene set ID: BRD-K02113016_olaparib_MCF7_24_h_10_um.

For reverse phase protein array (RPPA), BMDC were infected with RSV (MOI 2) for 2 h. Lysates were collected and sent to the RPPA core facility, MD Anderson Cancer Center, Texas, USA. Briefly, samples were treated with 1% sodium dodecyl sulfate (SDS) and beta-mercaptoethanol (B-Me). Proteins were arrayed on nitrocellulose-coated slides and probed with antibodies specific to the signaling molecules in their functional state (i.e., phosphorylation). Signals were captured by tyramide dye deposition and a DAB colorimetric reaction. Data were collected and quantitative analysis was performed for background correction, controlling for location, serial-dilution–signal-intensity curve construction, and concentration determination. The core provides normalized RPPA data in Log2 form (NormLog2) and data were analyzed for differential protein regulation (*p* value < 0.05) using the LIMMA statistical package in R.

### 2.8. Quantitative rt-PCR

Trizol reagent was used according to the manufacturer’s instructions (Invitrogen, Grand Island, NY, USA) to isolate the total RNA. Gene expression was analyzed using TaqMan Gene Expression Assay primer/probe sets on an ABI Prism 7500 Sequence Detection System (Applied Biosystems, Foster City, CA, USA). Custom primers were used to assess transcription levels of RSV-G, RSV-F, Muc5a, and Gob5, as previously described [32].

### 2.9. Real-Time Cell Metabolic Analysis

BMDC were infected with RSV for 2 h (MOI 2), washed thoroughly with Seahorse assay media (Agilent Technologies, Santa Clara, CA, USA), and incubated for 30 min in a CO_2_-free incubator at 37 °C. Total ATP production rates were measured according to the manufacturer’s instructions using the Seahorse XF Real-Time ATP Rate Assay Kit (Agilent Technologies, Santa Clara, CA, USA). Oligomycin (1.5 μM), Rotenone (0.5 μM), and Antimycin (0.5 μM) were used in the assay. Glycolysis rates were measured according to the manufacturer’s instructions using the Seahorse XF Glycolytic Rate Assay Kit (Agilent Technologies, Santa Clara, CA, USA). Rotenone (0.5 μM), Antimycin (0.5 μM), and 2-deoxy D-glucose (2-DG) (50 nM) were used in the assay. In each assay, 1 × 10^5^ cells were profiled and the Seahorse XFe96 Analyzer (Agilent Technologies, Santa Clara, CA, USA) was used to monitor the oxygen consumption rate (OCR) and extracellular acidification rate (ECAR). Data were further analyzed for interpretation using Seahorse XF Real-Time ATP Rate Report generator and Seahorse XF Cell Glycolysis rate Report generator.

### 2.10. Co-Culture Experiments

WT 129S or PARP1-/- BMDC were infected with RSV at a MOI of 1 or 2 for 4 h and then washed 2× with cold media. Naïve WT 129S mice were infected with RSV (2.5 × 10^5^) for 7 days, after which the lung draining lymph nodes were harvested. The tissue was enzymatically dispersed with collagenase A 1 mg/mL (Roche, Indianapolis, IN, USA) and 20 U/mL DNase I (Sigma, St. Louis, MO, USA) in RPMI with 10% FCS and further dispersed via an 18 gauge needle (10 mL syringe). RBCs were lysed and samples filtered through 100 micron nylon mesh. RSV responsive CD4+ T cells were isolated according to the manufacturer’s instructions using the EasySep™ Mouse Memory CD4+ T Cell Isolation Kit (STEMCELL Technologies, Toronto, ON, Canada). BMDC and CD4+ T cells were co-cultured (1:10 ratio) for 48 h, after which the supernatant was collected to measure cytokine protein levels using a customized Bioplex assay (Bio-Rad Laboratories, Hercules, CA, USA).

### 2.11. Statistical Analysis

Data were analyzed using Prism 7 (GraphPad Software); data are presented as mean values +/− SEM. Unpaired, two-tailed *t*-test was used to compare data between two groups. ANOVA was used to compare three or more groups. A *p*-value < 0.05 was considered statistically significant.

## 3. Results

### 3.1. RSV Differentially Regulates Key Transcriptomic and Metabolic Targets in Dendritic Cells

**Figure 1:**
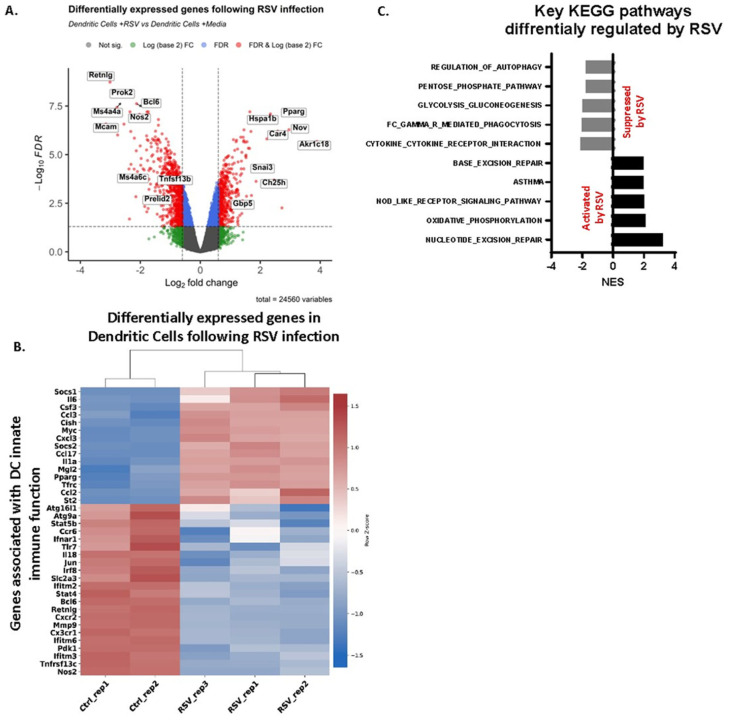
BMDC were infected with RSV for 24 h. (**A**) Volcano plot showing the totality of genes differentially expressed in DC following RSV infection (≥0.6 |log2FC| and FDR ≤ 0.05. (**B**) Cluster heatmap showing key genes involved in DC innate function; Z-scores were calculated for rows (genes). (**C**) Showing GSEA results (difference of class-RSV vs. Ctrl) of key KEGG pathways. RSV suppresses key pathways involved in metabolism and antiviral immune regulation. RSV activated DNA repair pathways which are known to facilitate induction of PARP1 enzymatic activity (FDR < 10%).

We performed global transcriptional analysis of bone-marrow-derived dendritic cells (BMDCs) infected with respiratory syncytial virus (RSV) (Figure 1). BMDC were infected for 24 h (Figure 1A–C). Over 1000 genes were differentially regulated in BMDC following RSV exposure (≥0.6 |log2FC| and FDR ≤ 0.05; Figure 1A). Focusing on key genes involved in DC innate immune function, RSV suppressed genes associated with DC antiviral functions (Figure 1B: Ifitm2/3, Tlr7, Nos2) while upregulating expression of pathogenic gene signatures (Figure 1B: St2, Ccl2, Illa). Gene set enrichment analyses (GSEA) indicated that the RSV activated DNA repair and specific pathways, including oxidative metabolism and NOD-like receptor signaling (Figure 1C). The pathway analysis also revealed that the global transcriptional signature induced in BMDC following RSV infection resulted in the suppression of pathways involving glycolytic metabolism, autophagy, and phagocytosis (Figure 1C). Together, these gene expression analyses provide interesting and clear data indicating an early and skewed gene expression response that promotes an inappropriate innate metabolic pathway program.

**Figure 2:**
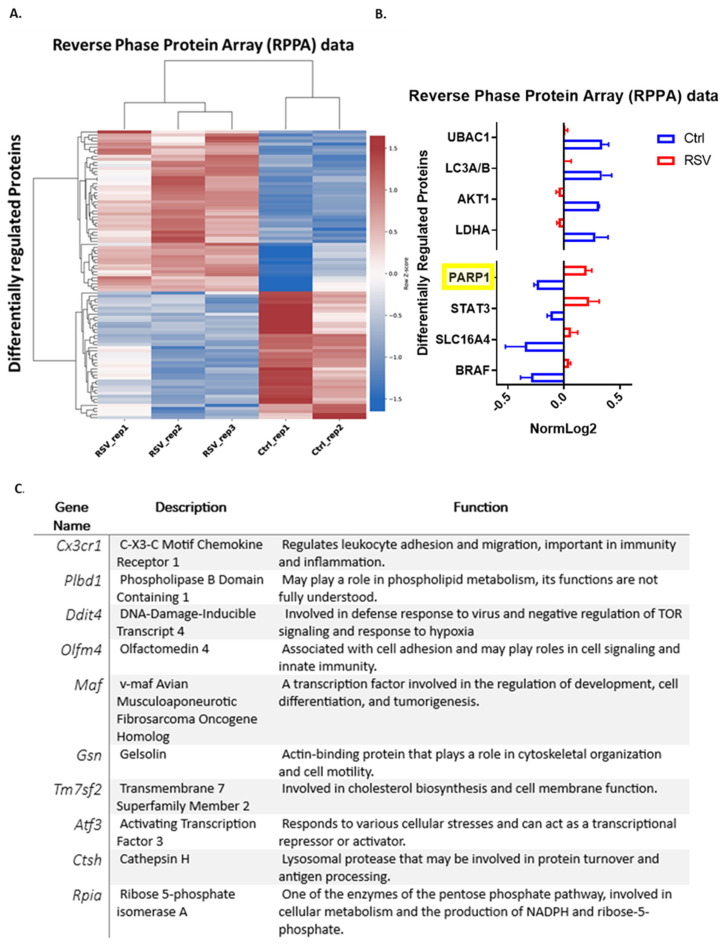
BMDC were infected with RSV for 2 h. (**A**) RPPA data showing key metabolism-associated proteins, including PARP1, as differentially regulated (*p* < 0.05) between the RSV and Ctrl conditions. RPPA identifies activating post-translational markers (i.e., phosphorylation) of cytosolic proteins. (**B**) Key metabolically relevant differentially regulated proteins. RPPA is a commercially available assay from the MD Anderson Cancer Centre, Texas, United States. Data presented as normalized log2 values (NormLog2). Values represent mean ± standard error of the mean (SEM). PARP1 was highlighted as the most differentially altered protein. (**C**) Transcriptionally downregulated genes in DC after RSV infection that are PARP1-modulated. The database of gene expression profiles for cell lines following pharmacological or genetic perturbation from the LINCS L1000 project was used to identify PARP1-modulated genes. Derived genes were then compared to the differentially regulated genes in DC following RSV infection.

Mitochondrial dysfunction leads to the release of mitochondrial metabolites (including mtDNA and mitochondria–nucleus associated proteins) into the cytosol, inducing oxidative and ER stress and exacerbating inflammation [33]. Post-translational protein modifications play a critical role in modulating immune–metabolic signaling pathways. To explore if these processes are linked, a quantitative assessment of key proteins was analyzed in cell lysates of uninfected and RSV-infected BMDC using a proteomics. RPPA is a proteomic technique that employs antibodies to simultaneously measure total protein abundances and post-translational modifications—in this assay phosphorylation events—enabling comprehensive profiling across numerous signaling pathways. RPPA analysis of DC showed a clear pattern of differentially phosphorylated proteins following 2 h of RSV infection, as illustrated by the heatmap in Figure 2A. Examining key proteins with metabolic contributions, the RPPA data show that metabolic signatures were differentially regulated in BMDC following RSV infection (Figure 2B). There was a significant decrease in the phosphorylation of autophagy (LC3A/B) and other key cell metabolism proteins (AKT1, LDHA), whereas PARP1 and STAT3 experienced the highest increase in RSV relative to uninfected BMDC. Given the novelty of PARP1 in the RPPA data, we further assessed transcriptional changes induced in DC by RSV related to PARP1 enzymatic activity. The upregulated genes in DC after RSV infection included base excision repair and nucleotide excision repair processes (Figure 1C), which are part of the DNA repair pathways that elicit PARP1 enzymatic activity. Furthermore, by using a defined PARP1-dependent dataset from the LINCS L1000 project [34] to identify PARP1 modulated gene sets, we found that a key subset of them were transcriptionally downregulated in DC following RSV infection (Figure 2C). Thus, PARP1 was shown to be activated by RSV at the protein level (Figure 2B), appearing as an interesting link between the transcriptomic metabolic signature and proteomic studies.

### 3.2. PARP1 Suppresses Key Elements of DC Innate Function during RSV Infection

**Figure 3:**
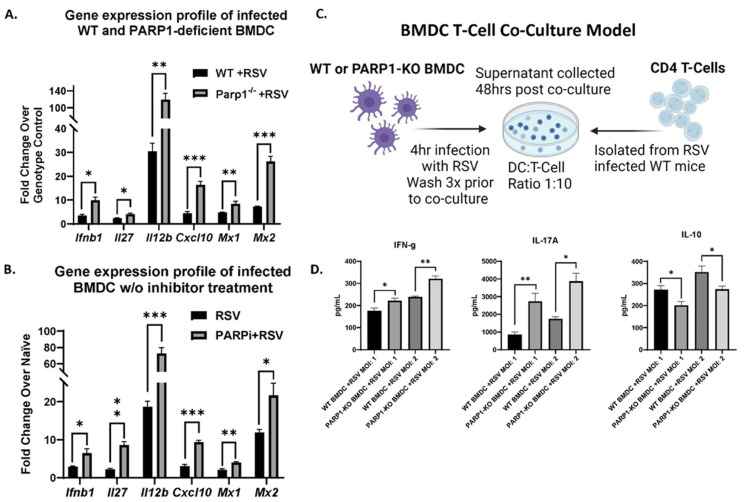
(**A**) mRNA expression of BMDC grown from WT 129S and PARP1-KO mice following 4 h RSV infection (N ≥ 5). (**B**) mRNA expression of BMDC grown from WT BALB/c mice treated with PARP1 inhibitor (PARPi), Talazoparib, for 30 min and then infected with RSV for 4 h (N ≥ 5). (**C**) BMDC/T-cell co-culture experimental design (created by BioRender.com): RSV-infected (MOI of 1 and 2) WT 129S or PARP1-KO BMDC co-cultured with CD4 T-cells isolated from the lymph nodes of RSV-infected WT 129S mice 8 days postinfection (**D**) Cytokines secreted in 48 h co-culture supernatants (N ≥ 5). Values represent mean ± SEM. *** *p* < 0.0005 ** *p* < 0.005 * *p* < 0.05.

Given that PARP1 appeared as a potential link between the two omics approaches, and given the novelty of targeting PARP1 during RSV infection, we sought to determine if its manipulation modulates DC innate function. BMDC were grown from PARP1-/- and WT mice, infected with RSV for 4 h, and then transcriptionally profiled via rt-PCR (Figure 3A). We also made use of an inhibitor treatment model using Talazoparib, a clinical phase PARP1 inhibitor. BMDC grown from WT mice were incubated with an inhibitor or a vehicle prior to a 4 h RSV infection and then transcriptionally profiled via rt-PCR (Figure 3B). Genomic ablation (Figure 3A) or enzymatic inhibition (Figure 3B) of PARP1-attenuated RSV-induced suppression of DC antiviral gene program were investigated. The PARP1-/- and inhibitor-treated BMDC were both able to upregulate Ifnb1, Mx1, and Mx2 gene expression in response to RSV infection. Type I IFNs (IFN) are potent pleiotropic cytokines that are critical to initiate a robust antiviral gene program but also strongly influence DC cellular metabolism [35,36,37]. Mx1 and Mx2 contribute to the suppression of viral replication and are both downstream of IFN induction [38]. Additionally, in the absence of PARP1, BMDCs were able to upregulate Cxcl10, Il12b, and Il27 gene expression in response to RSV (Figure 3A,B). These findings suggest that a lack of PARP1 would facilitate BMDC to elicit TH1 induction in response to RSV.

To determine whether the innate signature would impact DC function, we performed DC/T-cell co-culture experiments with infected PARP1-/- and WT BMDCs using T cells isolated from RSV-infected WT mice (Figure 3C). Using 4 h RSV infection of DC as the antigen presenting cell (APC) for activation of isolated RSV responsive T cells, co-culture experiments were established. Compared to WT, PARP1-/- BMDC drove a significantly higher production of IFN-γ and IL-17 protein production in T cells from RSV-infected mice (Figure 3D). Cytokine production from T cells increased with increasing multiplicity of infection (MOI) of BMDC, irrespective of genotype (Figure 3D). Additionally, PARP1-/- BMDC suppressed T cell production of IL-10 protein, with IL-10 being a negative regulator of TH1 processes [39] (Figure 3D). Thus, PARP1 blockade was found to lead to an overall increase in the antiviral innate immune profile of DC that can impact the activation of Th cell, enhancing Th1 cell skewing.

### 3.3. PARP1 Modulates Key Aspects of DC Metabolic Processes during RSV Infection

**Figure 4:**
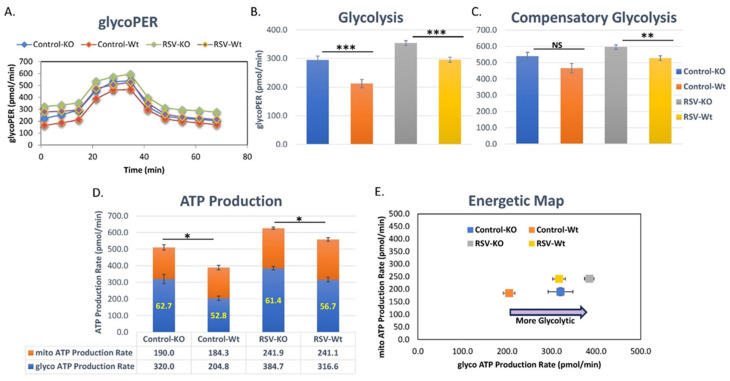
Real-time metabolic profile of BMDC grown from WT 129S and PARP1-KO mice following 2 h of RSV infection (N ≥ 5). (**A**–**C**) Glycolysis was measured using the Glycolytic Rate Assay Kit and analyzed with the Seahorse XFe96 analyzer. PARP1-KO BMDC were significantly more glycolytic at baseline and following RSV infection. PARP-KO BMDC also had a higher compensatory glycolytic rate following RSV infection. (**D**,**E**) The Seahorse XF Real-Time ATP Rate Assay Kit was used to measure ATP production from glycolysis and mitochondria simultaneously. Data were analyzed using the Seahorse XFe96 analyzer. With or without RSV infection, WT 129S and PARP1-KO BMDC had similar mitochondrial-derived ATP production rates. Abrogation of PARP1 significantly increased BMDC glycolysis-derived ATP production, resulting in higher net ATP levels compared to WT 129S BMDC following RSV infection. Values represent mean ± SEM. *** *p* < 0.0005 ** *p* < 0.005 * *p* < 0.05 NS—not significant.

PARP1 enzymatic activity is linked with significant depletion of cellular NAD pools, which are critical for various metabolic processes including glycolytic metabolism [40]. Immunometabolism is a key regulator of immune cell function and, in particular, glycolytic processes have been shown to influence DC innate function [41,42,43,44]. To investigate the influence of PARP1 in DC metabolism, we performed real-time metabolic profiling of control and RSV-infected BMDC derived from WT and PARP1-/- mice. The main contributors to extracellular acidification and proton efflux rates (PER) were CO_2_ molecules from glycolysis and mitochondrial respiration. The Seahorse-based Glycolysis Rate Test allows for the quantification of glycolysis-derived proton efflux rate (glycoPER) to measure the glycolytic rate of live cells. We found that, in the presence of normal PARP1 levels, there was a significant suppression of glycolysis in BMDC with or without RSV infection (Figure 4A,B). RSV-infected PARP1-/- BMDC had a significantly higher glycolysis rate than infected WT BMDC (Figure 4B). There was no difference in the compensatory glycolytic rate between uninfected WT and PARP1-/- BMDC (Figure 4C). Compensatory glycolysis refers to the overall glycolytic potential of cells following inhibition of mitochondrial respiration. Interestingly, in comparison to PARP1-/- BMDC, RSV significantly lowered the compensatory glycolysis of WT BMDC (Figure 4C). Overall, these data suggest that PARP1 suppresses the overall glycolytic capacity of the DC. This was made even more evident when assessing the overall energetic profile using the Seahorse Real-Time ATP Production Rate (Figure 4D,E). The mitochondrial-derived ATP production rate was consistent between WT and PARP1-/- BMDC in both infected and uninfected settings (Figure 4D). However, irrespective of RSV infection, the overall ATP production rate was significantly higher in PARP1-/- BMDC (Figure 4D). This difference in overall ATP production rate is explained primarily by the PARP1-/- BMDC having a significantly higher contribution of glycolysis-derived ATP (Figure 4D). The overall energetic map best summarizes these findings, with the metabolic profile of the PARP1-/- BMDC being more glycolytic in both the infected and uninfected settings (Figure 4E). Together, these data indicate an overall PARP1-associated regulation of DC innate phenotype that is linked to cellular metabolism, and the shift to glycolysis can be achieved in the absence PARP1.

### 3.4. PARP1-/- Mice Are Protected against RSV-Induced Immunopathology

**Figure 5:**
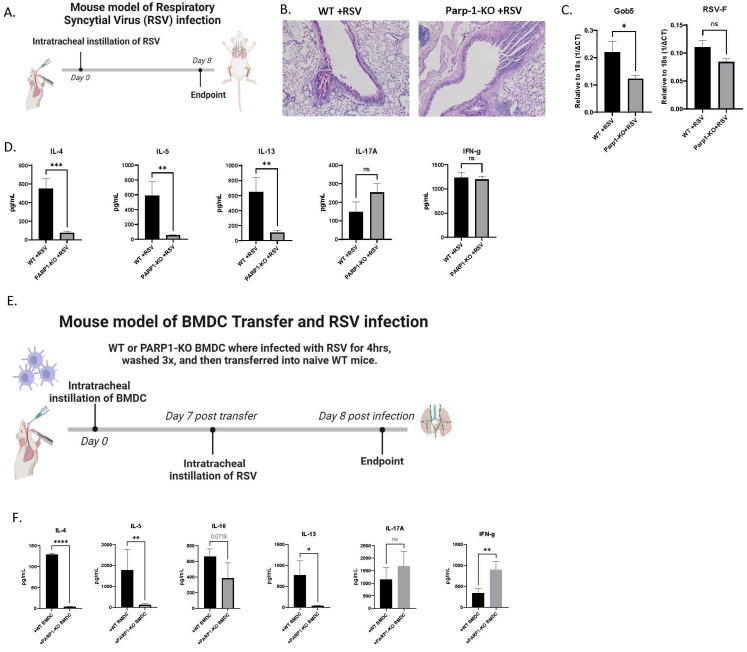
(**A**) Mouse model of RSV infection. WT 129S and PARP1-KO mice were infected with RSV via I.T. and harvested on day 8 postinfection. (**B**) Periodic acid-Schiff (PAS) staining of the lungs from WT 129S and PARP1-KO mice on day 8 post RSV infection. Representative photos shown (N ≥ 5). (**C**) Lung gene expression levels of Gob5. Values represent mean ± SEM. (**D**) LDLN were processed into single-cell suspensions and restimulated with RSV in vitro for 48 h to determine cytokine protein levels. Values represent mean ± SEM. (**E**) Mouse model of BMDC transfer and RSV infection. WT 129S and PARP1-KO BMDC were infected with RSV for 4 h, washed, and then transferred via I.T. into WT 129S mice which were then infected with RSV a week later. LDLN were harvested from recipient mice on day 8 postinfection. (**F**) LDLN cytokine protein data following 48 h postrestimulated with RSV. Values represent mean ± SEM. **** *p* < 0.00005 *** *p* < 0.0005 ** *p* < 0.005 * *p* < 0.05 NS—not significant.

Our in vitro and ex-vivo data suggest a strong role being played by PARP1 in driving RSV-induced innate and acquired immune responses. We made use of a mouse model of RSV infection using WT and PARP1-/- mice to assess the impact of RSV-induced PARP1 activation in driving viral-induced immunopathology. WT and PARP1-/- mice were administered RSV intratracheally (IT) and harvested on day 8 postinfection (Figure 5A). The histopathology showed that infected WT mice had more mucus staining than infected PARP1-/- mice (Figure 5B). Lung mRNA expression of Gob5, a mucus-associated gene, was significantly higher in infected WT vs. PARP1-/- mice, corroborating the histopathology (Figure 5C). The cytokine profile of the lung-draining lymph nodes (LDLN) of WT and PARP1-/- mice revealed significant regulation of acquired immune profiles. The supernatant from WT LDLN had significantly higher Th2 cytokine protein (IL-4, IL-5, and IL-13) compared to PARP1-/- LDLN following RSV infection (Figure 5D).

To highlight the role of dendritic cells in driving this phenotype, we made use of a BMDC transfer model of RSV infection (Figure 5E). BMDC were isolated from uninfected WT and PARP1-/- mice and then subjected to a 4 h RSV infection. These BMDC were washed and then transferred to the lungs of WT mice via I.T. After sensitization for a week (day 7 post-transfer), recipient mice were infected with RSV via I.T. and their LDLN were harvested on day 8 postinfection. Comparatively, cytokine profiling of the LDLN revealed that infected WT mice receiving PARP1-/- BMDC had significantly lower Th2 cytokine protein levels and higher IL-17A and IFN-γ (Figure 5F). These data, together, demonstrated two important aspects, (1) PARP1 deficiency promoted a protective antiviral phenotype and (2) DC alone appeared to be sufficient to drive the protective immune phenotype.

### 3.5. Depletion of PARP1 Enzymatic Activity Protected Mice from RSV-Induced Pulmonary Pathology

**Figure 6:**
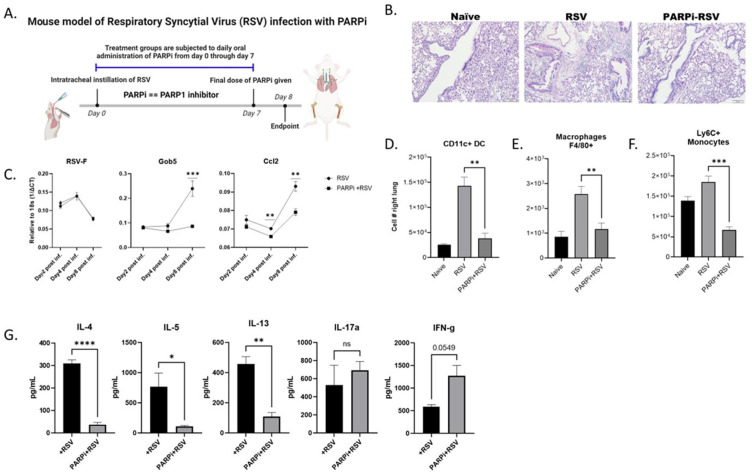
(**A**) Mouse model of PARP1 inhibitor (PARPi) treatment and RSV infection. WT BALB/c mice were infected with RSV and, 2 h later, treated with Talazoparib (0.33 mg/kg) or vehicle via oral gavage. The PARPi treatment was given daily through day 7 postinfection. Mice were harvested on day 8 postinfection (created by BioRender.com). (**B**) Lungs were embedded in paraffin, and PAS was performed to visualize mucus. Representative photos shown (N ≥ 5). (**C**) Lung gene expression levels of RSV-F, Gob5, and Ccl2. Values represent mean ± SEM. (**D**–**F**) Lungs were processed into a single-cell suspension, and flow cytometric analysis performed for cell number enumeration. Values represent mean ± SEM. (**G**) LDLN cytokine protein data following 48 h postrestimulation with RSV. Values represent mean ± SEM. **** *p* < 0.00005 *** *p* < 0.0005 ** *p* < 0.005 * *p* < 0.05 NS—Not significant.

Over the years, extensive research has gone into developing well-tolerated and highly effective PARP1 inhibitors. We made use of PARP1 enzymatic inhibition to further characterize the role of PARP1 in driving RSV-induced immunopathology. WT mice were infected with RSV and concurrently subjected to daily oral treatment with the PARP1 inhibitor Talazoparib (PARPi; 0.33 mg/kg) (Figure 6A). Talazoparib is a well-tolerated, clinical phase PARP1 inhibitor [45]. Histopathology showed that the airways of the PARPi-treated RSV-infected mice were free of mucus, while those of the RSV-only group exhibited significant mucus and were more occluded (Figure 6B). In agreement with the histology, the lung mRNA profile of these mice showed lower levels of Gob5 in the PARPi-treated infected group (Figure 6C). There was no difference in viral load (RSV-F) between infected groups (Figure 6C). Interestingly, PARPi treatment significantly lowered lung mRNA levels of the chemokine ccl2 (Figure 6C) and, correspondingly, the PARPi-treated mice had significantly lower infiltration of CCR2+ highly inflammatory cells (CD11C DC, macrophages, and monocytes) as measured by flow cytometry (Figure 6D). Cytokine profiling of the LDLN showed that PARPi treatment drove an increased production of IFN-γ protein (*p* value = 0.0549) and significantly reduced RSV-driven Th2 cytokine induction (Figure 6G). Thus, treatment of WT mice with the clinically approved PARP1 inhibitor allowed abrogation of RSV-induced pathology and an antiviral immune phenotype.

### 3.6. Delayed/Therapeutic PARPi Treatment Has an Incomplete Effect

**Figure 7:**
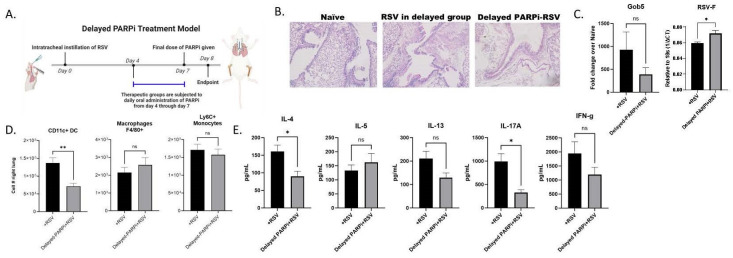
(**A**) Therapeutic mouse model of PARPi treatment and RSV infection. WT BALB/c mice were infected with RSV. Mice were subjected to daily treatment with Talazoparib (0.33 mg/kg) or vehicle via oral gavage from day 4 through to day 7 postinfection. Mice were harvested on day 8 postinfection (created by Biorender.com). (**B**) Lungs were embedded in paraffin and PAS staining was performed to visualize mucus. Representative photos shown (N ≥ 5). (**C**) Lung gene expression levels of Gob5. Values represent mean ± SEM. (**D**) Lungs were processed into a single-cell suspension, and flow cytometric analysis performed for cell number enumeration. Values represent mean ± SEM. (**E**) LDLN cytokine protein data following 48 h of postrestimulation with RSV. Values represent mean ± SEM. ** *p* < 0.005 * *p* < 0.05 NS—not significant.

The findings from our in vivo approaches suggest that the PARPi treatment could be a viable therapeutic strategy for addressing the immunopathology triggered by RSV. To further test its appropriateness for clinical application, a therapeutic mouse model of PARPi treatment and RSV infection was used (Figure 7A). Given that hospitalization with RSV tends to occur several days postinoculation, the PARPi treatment was delayed until day 4 postinfection. Mice were harvested on day 8 postinfection to assess the immunopathology. There were no clear differences in the histopathology between groups (Figure 7B), while lung mRNA levels of Gob5 were found to have decreased but not significantly in the infected delayed treatment group (Figure 7C). Flow cytometry analysis showed that the lungs of individuals in the infected delayed treatment group had significantly lower infiltration of CD11c+ DC, with no difference in macrophage or monocyte levels (Figure 7D). Cytokine profiling of the LDLN revealed that only IL-4 and IL-17A protein levels were significantly reduced in the delayed treatment group following RSV infection (Figure 7E). These data suggest that, in the context of RSV infection, the effects of PARPi treatment are more impactful on the innate compartment and support the previous data that an early modification of the DC/APC function may be most important for modifying the immunopathology.

## 4. Discussion

Dendritic cells (DC) are one of the most important immune cell types, as they guide antigen presentation and innate cytokine secretion to dictate the nature of the immune response to various disease-producing agents. Several viral pathogens, including RSV, manage to suppress robust induction of type I interferons (IFN), which are needed to drive the appropriate antiviral Th1 immune state [46,47,48]. In response to toll-like receptor (TLR) stimulation, DC undergo robust induction of glycolytic metabolic reprograming to allow the activation of a type I IFN gene program [42,44,49]. Type I IFN facilitate a continued commitment to this metabolic state through the NOS2 axis, a process dependent on the NAD+ metabolome [50]. The metabolites from glycolysis and the associated pentose phosphate pathway are used in various biosynthetic pathways, and suppression of glycolic metabolism in DC has been shown to drive innate immune dysfunction and enhance viral replication [41,51,52,53]. This suggests an intimate link between cellular metabolism and establishment of an innate immune-cell-driven antiviral state. Recent studies have linked RSV to manipulation of fatty acid synthesis, increases in oxidative stress, and mitochondrial dysfunction [28,54,55,56,57,58]. Inhibiting FASN or ACC1 protects both epithelial cells and DC against RSV-induced cytopathology [28,58]. Potentially, downstream of RSV-induced oxidative stress and increases in fatty acid synthesis are part of the activation of the DNA damage response element PARP1, as observed with our RPPA data. The enzymatic function of PARP1 involves auto/trans deposition of poly-ADP-ribose units, an NAD+-dependent process that can regulate numerous transcriptional programs directly and indirectly [59]. It appears that PARP1 activation impacts cellular metabolism primarily through acute depletion of cellular NAD+ levels [22,23]. Additionally, PARP1 directly suppresses glycolytic metabolism through PARylation of HK1, a rate limiting enzyme [60]. NAD+ has various roles in a multitude of cellular processes, with the vast majority sequestered in the mitochondria [61]. PARP1-mediated depletion of NAD+ drives mitochondrial dysfunction and suppresses the activities of SIRT1, an NAD+-dependent enzyme important for metabolic homeostasis as well as autophagy [62,63]. Our data demonstrates two main consequences of RSV-mediated PARP1 activation: (1) alteration of DC cellular metabolism during the early phase of infection results in the suppression of antiviral properties and (2) enhancement of viral-induced pulmonary pathology includes airway inflammation and mucus hypersecretion. The DC-T-cell co-culture studies and delayed infection models strongly suggest that the T cell response changes are a consequence of the DC regulation that is central to directing immune response outcomes. Disruption of host metabolism is a key component of the cytopathology induced during viral infections and RSV, in particular, targets DC cellular metabolism to drive systemic pathology.

The real-time cell metabolic analysis data demonstrate that genomic ablation of Parp1 shifts DC metabolism toward an enhanced glycolytic process. In the absence or blockade of PARP1, RSV was able to induce glycolysis more effectively in DC, further suggesting that PARP1 facilitates inappropriate metabolic pathways. Genomic ablation or enzymatic inhibition of PARP1 is linked to the protection against fatty liver disease, increases in NAD+ levels, increased glycolysis, and reduced mitochondrial damage [60,63,64,65,66]. While our studies have not fully evaluated mitochondria health, the shift toward glycolysis suggests a better innate antiviral immune response as indicated by enhanced type I IFN and IL-12. Various cellular processes including the L-arginine–NOS and glycolysis pathways are heavily reliant on the NAD metabolome. In fact, NAD depletion induces mitochondrial dysfunction and impairs autophagy-related pathways which are induced in RSV-infected DC and necessary for antiviral innate cytokine production [40,61,63,64,67]. Previous work in our laboratory has shown that the autophagy-linked histone deacetylase SIRT1 is protective against RSV-induced aberrant DC metabolism, and this occurs through two distinct processes. First, SIRT1 regulates autophagy which is critical to DC-mediated RSV antigen processing and, second, SIRT1 facilitates mitochondrial homeostasis. PARP1-mediated depletion of cellular NAD pools antagonizes these protective aspects of SIRT1 activity, with several studies showing that PARP1 depletion improves mitochondrial integrity and function. Furthermore, PARP1 enhances both STAT3 activity and expression levels [68]. Also enhanced by RSV in our studies, STAT3 is a negative regulator of DC antiviral innate immune function, in part due to facilitating-increased IL-10 production [69,70,71,72]. While our studies have not linked PARP1 and STAT3 activation, previous publications have linked STAT3 to cell metabolism, likely playing a significant role as well [73,74]. Additional studies are needed to specifically address the role of STAT3 in RSV pathogenesis.

**Figure 8:**
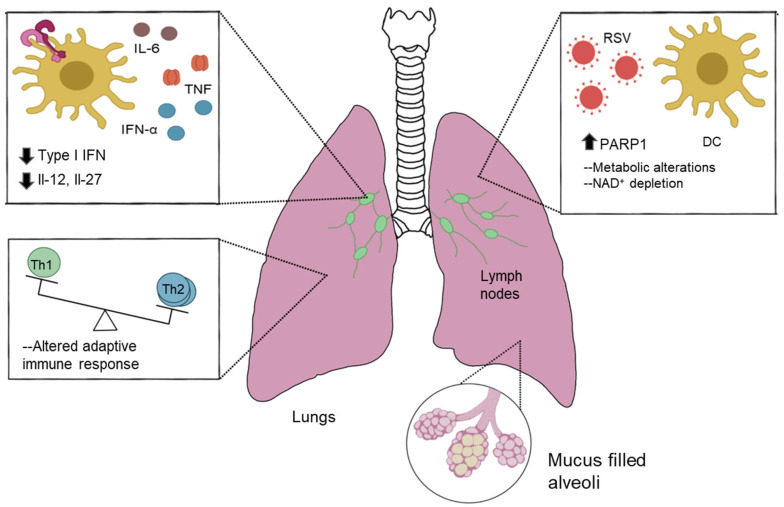
Proposed effect of RSV-induced PARP1 in DC-mediated antiviral responses. RSV drives induction of pathways facilitating PARP1 activity. PARP1 facilitates perturbed cellular metabolism, including depletion of NAD+, driving pathologic inflammatory responses and suppressing DC-mediated antiviral function.

The metabolic switch seen with the stimulated DC is crucial to facilitate the induction of the innate functions and the eventual mobilization of the immune system toward an antiviral, nonpathogenic response. Our data show that RSV-induced PARP1 activity in DC promotes a more pathogenic environment through dysregulation of cellular metabolism, resulting in a marked suppression of key type I IFN genes. PARP1 enzymatic inhibition drives the amelioration of Th2-mediated inflammation without affecting the antiviral elements of Th1-mediated processes, therefore mitigating the most detrimental aspects of the disease pathogenesis. Additionally, intermediate metabolites from the glycolytic pathway and the associated pentose–phosphate pathway are used in various biosynthetic pathways, with the inhibition of the type-I-IFN-induced Warburg state in DC facilitating enhanced viral replication [41,43,55]. RSV-induced PARP1 activity functions to perturb key features of cellular metabolism such as the glycolytic and pentose–phosphate pathways that depend on the NAD+ metabolome. In addition to metabolic changes, the overall mechanism for PARP1 is likely to be complicated owing to its ability to modulate transcription factors and chromatin structures, areas that will be investigated in the future. Overall, our data strongly suggest that PARP1 has a pivotal role in DC innate cell function through metabolic reprogramming leading to a limited antiviral gene program and provide potential therapeutic avenues for PARP1 inhibition (Figure 8).

## Data Availability

We are in the process of depositing all data from the RNA analysis used in this manuscript in the NIH GEO database. Prior to the complete upload we can share data directly upon request.
